# Exploratory analysis of smartphone-based step counts as a digital biomarker for survival in ALS patients

**DOI:** 10.3389/fdgth.2025.1705368

**Published:** 2026-01-29

**Authors:** Marcos Matabuena, Marcin Straczkiewicz, Narghes Calcagno, Katherine M. Burke, Timothy B. Royse, Amrita Iyer, Kendall T. Carney, Sydney Hall, James D. Berry, Jukka-Pekka Onnela

**Affiliations:** 1 Department of Biostatistics, Harvard T.H. Chan School of Public Health, Boston, MA, United States; 2 Department of Measurement and Electronics, AGH University of Krakow, Krakow, Poland; 3 Department of Neurology and Laboratory of Neuroscience, IRCCS, Istituto Auxologico Italiano, Milan, Italy; 4 Neurological Clinical Research Institute and Sean M. Healey & AMG Center for ALS, Massachusetts General Hospital, Boston, MA, United States; 5 School of Health and Rehabilitation Sciences, PhD in Rehabilitation Sciences Program, MGH Institute of Health Professions, Boston, MA, United States

**Keywords:** amyotrophic lateral sclerosis, digital biomarkers, smartphone, accelerometry, physical activity, survival analysis, distributional data analysis

## Abstract

Amyotrophic lateral sclerosis (ALS) is a progressive and debilitating neurodegenerative disease. Digital biomarkers derived from smartphone data can enable scalable, low-cost, remote, unobtrusive, and quantitative measurement of physical activity (PA). These biomarkers offer opportunities for quasi-continuous assessment of PA levels, which may provide new methods for monitoring ALS disease progression in real time. In this exploratory study, we analyzed data from 31 individuals with ALS (including 16 deaths) with up to 9 years of follow-up (median 3 years) to assess the impact of incorporating smartphone-derived PA measures into survival prediction models. We examine whether the strength of the statistical association with survival differs when PA is summarized as (i) a simple metric, such as the mean daily step count, vs. (ii) distributional representations of PA. The exploratory results suggest that the addition of PA variables defined via distributional representations improves the performance of the model, as reflected by higher C-score values (0.68 vs. 0.55, estimated as the median over bootstrap replicas B=1,000). A bootstrap-based hypothesis test shows statistically significant differences between the two models at the confidence level of 90%. These exploratory results indicate that the use of more advanced metrics to summarize PA time series can produce more accurate digital biomarkers to monitor the progression of ALS, although larger studies with larger sample sizes are required to confirm these findings.

## 1 Introduction

Amyotrophic lateral sclerosis (ALS) is a progressive neurodegenerative disease with marked heterogeneity in clinical course and limited disease-modifying options [1]. Monitoring in ALS trials has traditionally been based on functional scales, quantitative strength tests, and measures of lung function; the revised ALS Functional Rating Scale (ALSFRS-R) and its self-entry version (ALSFRS-RSE) remain the standard outcomes for tracking functional decline [2–4].

Ubiquitous smartphones and wearables now enable scalable, low-burden capture of real-world behavior and motor function, opening the door to *digital biomarkers* collected in free-living settings [5–7]. These data streams can support more frequent assessments, reduce participant burden and costs, and potentially accelerate therapeutic development [8]. Prior work has demonstrated that passive movement and activity measures correlate with conventional ALS endpoints [7, 9], and early evidence from wearable sensors suggests associations with disease progression and survival [10] However, the prognostic value of *smartphone-derived* physical activity (PA)–particularly when modeled beyond simple averages—remains underexplored.

We hypothesized that modeling the *distribution* of daily smartphone-based step counts over a short baseline window would capture clinically relevant variability (e.g., low- vs. high-intensity days) that is missed by mean-only summaries. Leveraging quantile function representations of daily step-count distributions and summarizing them via functional principal components, we evaluate the added value of smartphone PA for survival prediction in ALS. Specifically, we compare (A) a baseline model using age and ALSFRS-RSE, (B) an extended model that adds mean daily steps, and (C) a distributional model that adds quantile-based PA profiles. Our primary endpoint is the Concordance index (C-score) to quantify prognostic discrimination.

## 2 Methods

### 2.1 Ethical approval

The study protocol was approved by the Mass General Brigham Institutional Review Board. Written informed consent was obtained from all participants prior to enrollment.

### 2.2 Study design and reporting standards

This observational study adheres to the Strengthening the Reporting of Observational Studies in Epidemiology (STROBE) guidelines.

### 2.3 Data collection

Data used in this analysis were collected in three single-site observational studies conducted between 2016 and 2022 at the Sean M. Healey & AMG Center for ALS at Massachusetts General Hospital. All three studies followed a longitudinal, observational and non-interventional design. This manuscript reports a retrospective analysis of participants enrolled during this period, using previously acquired data to address new research questions.

The participants installed the Beiwe application on their iOS or Android smartphones. A total of 19% of the participants used Android devices, and no technical differences between the operating systems are expected to affect the final results. Beiwe is the front-end of a platform designed to capture data from smartphone sensors and patient-reported outcomes, upload these data securely to the cloud, and facilitate analysis via a library of statistical methods tailored to raw smartphone data. Participants were ambulatory at the beginning of the study and self-reported regular use of smartphones. They were instructed to use their smartphones as usual throughout the study.

For the purposes of this work, we focused on (1) passively collected data from smartphone accelerometers to quantify physical activity (PA) and (2) actively collected data by completing the ALSFRS-RSE [3] within the smartphone application. The ALSFRS-RSE is the self-entry version of the ALS Functional Rating Scale–Revised (ALSFRS-R). ALSFRS-R includes 12 items, each scored on a scale of 0 to 4, with lower scores reflecting lower functional status. Items’ scores are summed to generate a total score ranging from 0 to 48. Four subdomains–bulbar, fine motor, gross motor and respiratory [2] by adding the three elements within each domain. In this study, ALSFRS-R was embedded directly in the smartphone application and participants completed each item independently, without the help of the study staff. The self-entry version (ALSFRS-RSE) has been shown to be highly correlated with traditional staff-administered ALSFRS-R, supporting its validity for remote self-report.

Each study consisted of 3, 6, or 12 months of Beiwe data collection. The survival data was retrospectively collected by study staff in February 2024 by reviewing the medical and public records of all participants.

### 2.4 Statistical methods for distributional physical activity data representations

Wavelet-based approaches for estimating mean daily step counts and minute–by–minute step counts based on raw smartphone accelerometer data have previously been described [5–7, 11] and were used to quantify PA for this analysis. Step counts and wear time were estimated from smartphone accelerometer data following previously published methods [9]. A variable used in this analysis was calculated as the ratio between daily step counts and wear time (and expressed as steps per hour). In all analyzes in this paper, we use as a reference variable the ratio of daily step counts to daily wear time (in hours).

Following our previous distributional data analysis work [12, 13], for a participant i, i=1,…,n, we let $Y_{i}(t)$ denote the estimated time series of the daily step count, $t\in S_{i}$, where $S_{i}$ is the set of days with valid PA data. To minimize the impact of missing data, a day was considered “valid” if at least 4 h of accelerometer data were collected. The distributional representations of physical activity (PA) were then generated using valid days within 60 days of enrollment, and participants were required to have at least 14 valid days of data. Although these missing-data criteria are stringent, we consider this conservative choice appropriate for an exploratory study aimed at obtaining reliable distributional summaries of physical activity. With fewer than 10 days of data per participant, we cannot reliably estimate an individual’s activity distribution, whereas using much longer time windows (e.g., beyond 60 days) would implicitly assume stationarity and therefore risk bias due to distributional changes driven by ALS progression. Our previous analysis of this cohort [Karas et al. [11]] showed that missingness was not meaningfully associated with demographic characteristics in this data set and reported activity plots for several participants to illustrate the temporal heterogeneity of missing-data patterns. Together, these considerations support the use of conservative inclusion criteria that prioritize the reliability of our exploratory analyzes over maximizing the sample size.

For each participant i, we estimate the probability of taking fewer than s steps per day using the empirical distribution function of step counts

$${\hat{F}}_{i}(s)=\frac{1}{\left|S_{i}\right|}\sum_{t\in S_{i}}1\{Y_{i}(t)\leq s\}.$$

For each probability level p∈[0,1], we define the corresponding empirical quantile function

$${\hat{Q}}_{i}(p)=\min\{s\;:\;F_{i}(s)\geq p\},$$

which serves as a distributional representation of PA. This function specifies the minimum number of steps s for the individual to have more than or equal to p to take at least this number of daily steps. From a practical analytical perspective, we approximate each quantile function on an equispaced grid of 11 points in the domain [0,1], $p_{m}=\left\{\frac{m}{10}\;:\;m=0,1,\cdots ,10\right\}$. No additional smoothing is applied to these discretized quantile representations. For each participant i, we consider the Cox proportional hazards model

$$h_{i}(t)=h_{0}(t)\exp\;\left\{\beta_{\text{Age}}\;{\text{Age}}_{i}+\beta_{\text{FRS}}\;{\text{FRS}}_{i}+\sum_{\;j=1}^{6}\beta_{\text{pc}j}\;{\hat{\text{pc}}}_{ij}\right\},$$

where $h_{i}(t)$ is the participant-specific hazard function. The quantile function of physical activity, ${\hat{Q}}_{i}(\cdot )$, is evaluated on a grid $0\leq p_{0}<\cdots <p10\leq 1$, and summarized by the scores ${\hat{\text{pc}}}_{ij}$, j=1,…,6, obtained through the functional principal component analysis (FPCA) of discretized values ${\hat{Q}}_{i}(p_{m}){m=0}^{10}$.

As is commonly done, the number of scores in the FPCA method was selected based on a general rule of thumb. In our case, six components captured 98% of the total variability in the quantile function profiles of the step count.

In order to evaluate the predictive capability of the different Cox survival models, we consider the Concordance Index (C-score) metric, defined as

$$\text{C-score}=P(T_{i}>T_{j}|X_{i}>X_{j}),$$

where $T_{i}$ and $T_{j}$ are the observed survival times of the participants i and j, respectively, and the expression specifies the probability that the event $T_{i}>T_{j}$ occurs given that $X_{i}>X_{j}$. The terms $X_{i}$ and $X_{j}$ are the linear predictors of the Cox model for participants i and j. In our setting,

$$X_{i}=\beta_{\text{Age}}\;{\text{Age}}_{i}+\beta_{\text{FRS}}\;{\text{FRS}}_{i}+\sum_{k=1}^{6}\beta_{{\text{PC}}_{k}}\;{\hat{\text{PC}}}_{ik}.$$

The sign and magnitude of the Cox model coefficients determine whether each score is positively or negatively associated with the survival time. In general, the C-score measures the rank correlation between the predicted risk scores and the observed survival times: a C-score of 0.5 indicates that the predictive capacity is not beyond random chance, while a C-score of 1 indicates perfect prediction. To avoid overfitting when reporting pointwise C-score estimates, we assess the stability of the estimated C-score between bootstrap resamples B=1,000 (with replacement) and report the median over bootstrap replicas. We inspect the resulting distributions graphically using boxplots, and we use the empirical distribution of the bootstrap C-scores to quantify the statistical uncertainty of each model. In a setting like ours, with a limited sample size, the use of the bootstrap is expected to improve both the calibration of point estimates and the quantification of statistical uncertainty.

Finally, we note that FPCA is applied to the estimated quantile functions of daily physical activity (PA), rather than directly to the raw daily PA time series. The purpose of this exploratory analysis is to evaluate statistical associations with survival rather than to provide a detailed substantive interpretation of these associations. In general, coefficients of the scalar-on-function regression models based on quantile functions are difficult to interpret because the quantile function is defined at the individual level: the value of a given probability p∈[0,1] typically corresponds to very different values of the variable interest between individuals. Our main goal in using FPCA is therefore to obtain a low-dimensional representation of the data; given the small sample size, we do not focus on detailed interpretability of the resulting functional components of the quantile functions. To obtain more interpretable information, we also performed a cluster analysis of the estimated quantile trajectories of PA and stratified the individuals into three activity groups (high, moderate and low activity) and then examined the mortality rate within each of these groups. We perform a clustering analysis with K=3 groups applying the k-means data algorithm to FPCA scores.

### 2.5 Survival analysis details

All statistical analyses were performed in R. Cox models were fitted using the survival package and scores were extracted with functional principal component analysis (fPCA) using the fda package.

To assess the impact of PA on survival, we specify three Cox regression models to compare prediction accuracy:

1. **Model A:** Total score of ALSFRS-RSE + age
2. **Model B:** ALSFRS-RSE total score + age + average daily step counts (first 60 days)
3. **Model C:** ALSFRS-RSE total score + age + distribution of daily step counts (using quantile-based functional profiles)

We used quantile functions as distributional representations to capture low- and high-intensity PA days (i.e., lower and higher quantiles, respectively) and other distributional patterns of physical activity, such as day-to-day variability (Model C). We compared this with the use of traditional scalar summaries of PA that focus solely on average values and may discard relevant information on variability over days in physical activity patterns and other distributional characteristics (Model B).

## 3 Results

Eighty-two participants were enrolled, of whom 60 (73%) were male. The average age was 57 years (SD = 9.7; range 34 to 76). The mean baseline ALSFRS-RSE score was 34 (SD = 8.7). The mean diagnostic delay (time between the onset of the ALS symptom and the diagnosis of ALS) was 24.3 months (SD = 18.2).

**Figure 1:**
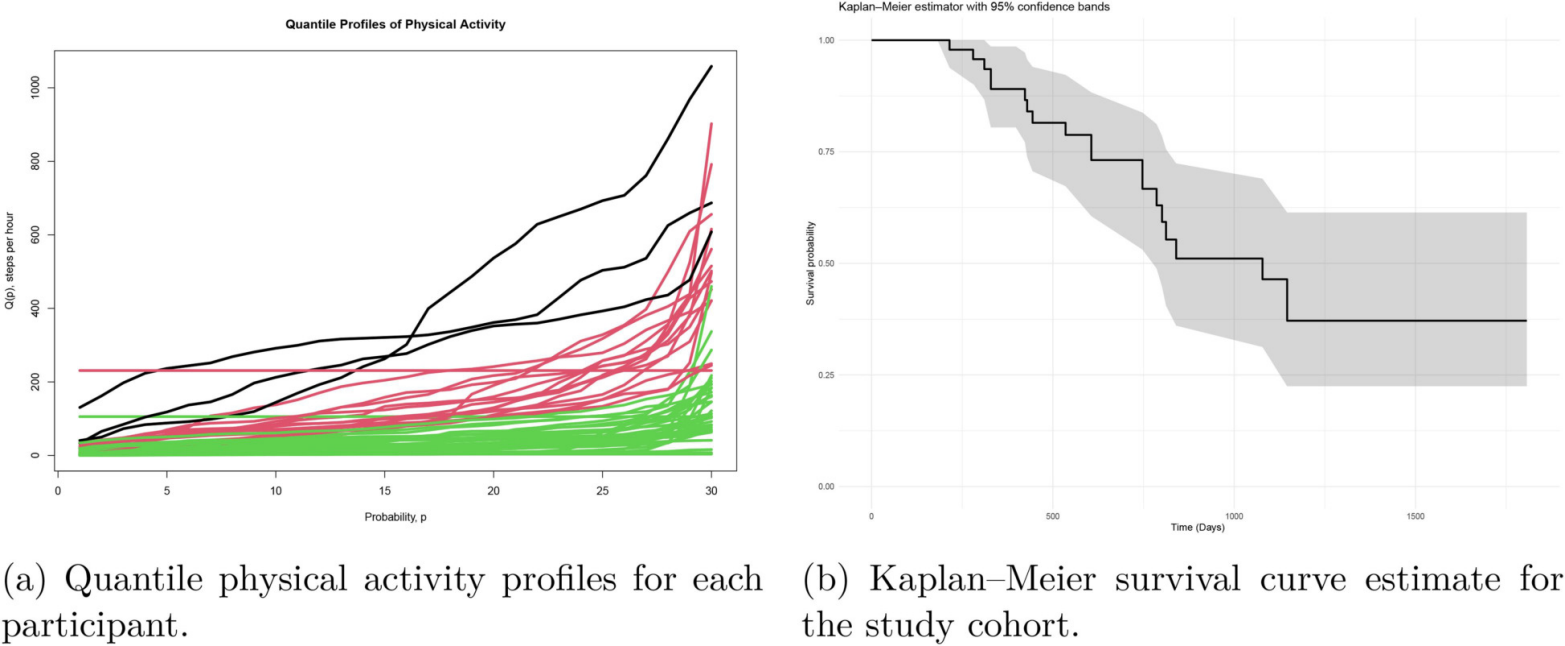
**(a)** Quantile physical activity profiles for each participant after preprocessing, using daily step counts normalized by wear time (in hours). The *x*-axis represents the probability p∈[0,1] of the quantile function, and the *y*-axis represents the evaluation of the quantile function Q(p). Individuals were stratified into three physical activity clusters (high, moderate, and low activity), with the corresponding trajectories highlighted in black, red, and yellow, respectively. **(b)** Kaplan–Meier survival curve estimate for the study cohort. The *x*-axis indicates time t in months, and the *y*-axis the survival probability. The follow-up time ranges up to 9 years, with an estimated median survival time of around 3 years. Pointwise 95% confidencee bands for survival functions are displayed.

Participants were included in the survival analysis if they had valid PA data (at least 14 days with 4 h of wear time during the first 60 days after enrollment) and completed ALSFRS-RSE at baseline. Thirty-one of the 82 participants met these criteria and were included in our analysis. Of these, 16 died at the end of the follow-up period. Because participants enrolled in the study at different time points, they had variable follow-up durations, ranging from 1 to 9 years. Among participants who died, 90% did so within the first three years of follow-up. The follow-up time was at least three years for 86% of the overall cohort (including those still alive). Figure 1a shows the Kaplan–Meier survival function estimate together with the 95% confidence bands. The estimated median survival time is at least 3 years; beyond this point, the remaining participants can be considered long-term survivors.

We compared the demographic and clinical characteristics of the analyzed cohort with the full cohort using the Kolmogorov–Smirnov test and found no statistically significant differences. This suggests that the analyzed group is representative of the overall cohort.

There are distinct patterns of low-intensity (quantiles p<0.3), mid-intensity (0.3<p<0.8) and high-intensity (p>0.8) PA among participants [Figure 1b, individual quantile functions of PA derived from step count functions]. The largest differences between individuals appear in the high-intensity range (p>0.8). Figure 1b shows, in black, red and yellow, the trajectories belonging to the high, median, and low-activity groups, respectively. The corresponding mortality rates are 33%, 50%, and 70%, indicating that ordering subjects according to their high-intensity quantile functions via intensity-based clustering effectively stratified mortality risk.

Table 1 presents the estimated coefficients (expressed as hazard ratios) for each variable, together with their 95% confidence intervals and *p*-values. We incorporated the functional profiles into the Cox model through principal component analysis, with the resulting scores being used as covariates in the survival model. Our analysis shows that Model C, which includes quantile-based PA measures, outperforms the other two models, substantially strengthening the statistical association compared to Model A and Model B (C-scores of 0.54, 0.55, and 0.68, respectively). In contrast, Model B incorporates average step counts, disregarding distributional patterns and variability throughout the 60-day observation period, and therefore provides only a 2% improvement in prediction compared to Model A without PA.

**Table 1:** Model coefficients, statistical significance, and predictive capacity. Model A includes age and baseline ALSFRS-RSE total score as predictors. Model B includes age, baseline ALSFRS-RSE total score, and average step counts per hour. Model C includes age, baseline ALSFRS-RSE total score, and the quantile step-count profile as predictors. The variables Scores 1–6 summarize the quantile physical activity (PA) trajectories for each participant. The table reports hazard ratios (HRs), 95% confidence intervals (CIs), C-score, and *p*-values for Models A–C for testing the null hypothesis that the hazard ratio is equal to 1. The C-score is calculated as the median of B=1,000 bootstrap replicates.

Variable	Hazard ratio	95% CI (HR)	*p*-value
Model A			
Age at baseline	1.02	0.99–1.05	0.22
Baseline ALSFRS-RSE	0.98	0.93–1.03	0.42
C-score	0.54		
Model B			
Age at baseline	1.03	0.99–1.08	0.19
Baseline ALSFRS-RSE	0.98	0.94–1.02	0.30
Average step counts per hour	1.00	0.93–1.07	0.77
C-score	0.55		
Model C			
Age at baseline	1.01	0.90–1.14	0.87
Baseline ALSFRS-RSE	0.99	0.94–1.04	0.70
Scores1	1.00	0.94–1.06	0.59
Scores2	1.00	0.93–1.07	0.67
Scores3	1.01	1.00–1.02	0.16
Scores4	0.98	0.96–1.00	0.02
Scores5	1.00	0.92–1.11	0.92
Scores6	1.03	0.97–1.09	0.31
C-score	0.68		

**Figure 2:**
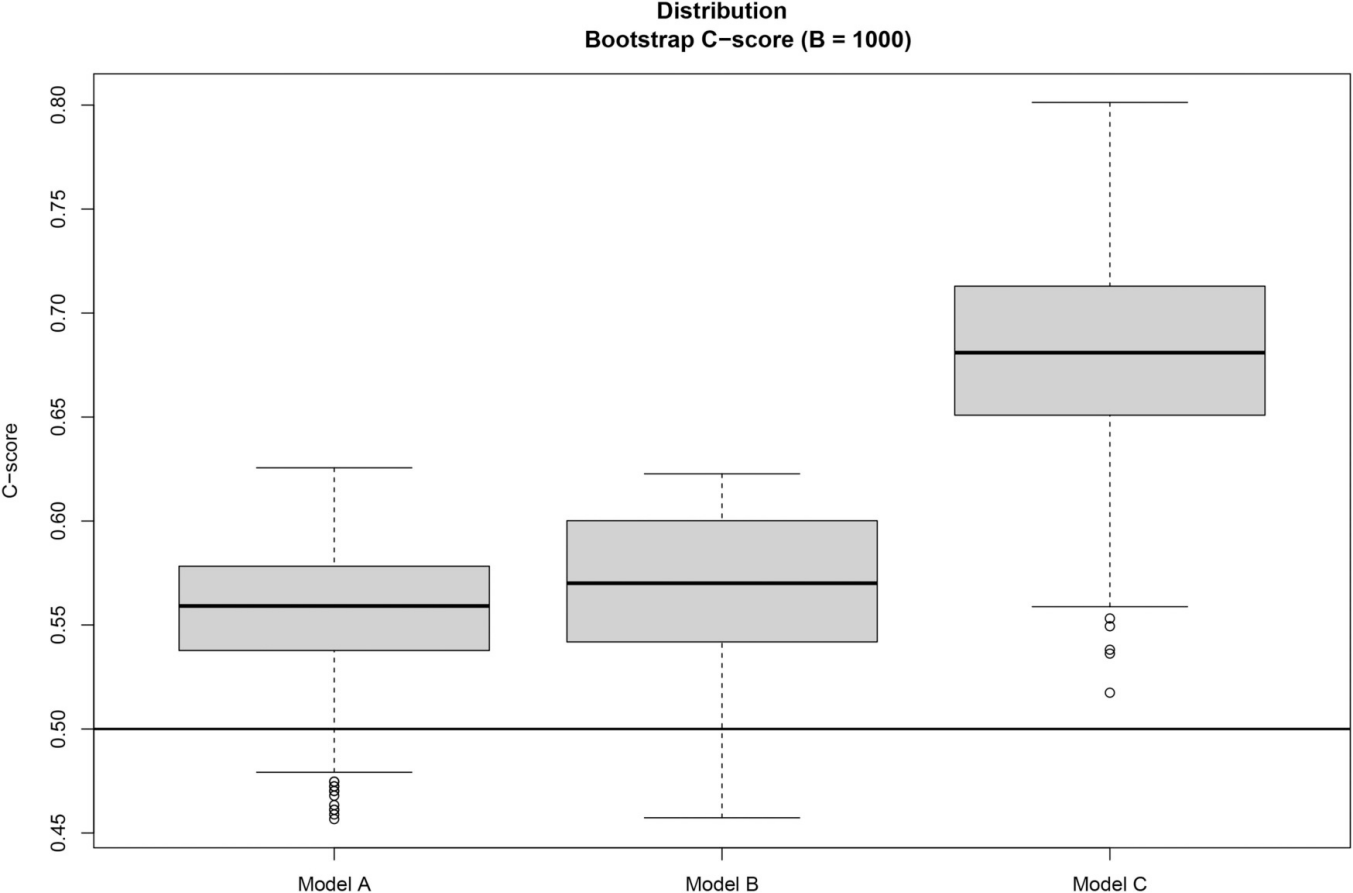
Distribution of the C-score statistical association metric for models A, B, and C across B=1,000 bootstrap replicates. Based on the bootstrap results, Model C outperforms Models B and A, with statistically significant differences in predictive performance at least at the 90% confidence level.

Finally, Figure 2 displays box plots of the C-score obtained from replacement bootstrap samples B=1,000, which quantify the uncertainty and variability of the C-score estimates across models A, B and C. Based on the bootstrap distributions, model C outperforms models A and B with at least 90% confidence, despite the small sample size of this exploratory analysis, while the differences between models A and B are minimal.

These results suggest that capturing the full distributional characteristics of physical activity is necessary to improve the predictive performance of the model and retain useful clinical information, since two individuals can have the same average level of physical activity, but differ in variability patterns or in other quantiles.

## 4 Discussion

The purpose of this study was to examine the role of physical activity (PA) measurements—collected through smartphone devices in free-living settings—in predicting survival in people living with ALS using two methods to quantify PA. Our findings suggest that incorporating physical activity (PA) patterns via distributional representations improves survival prediction compared to simpler models that include only age and baseline ALSFRS-RSE scores, with gains in predictive performance that are statistically significant at the 90% confidence level. These results also highlight the additional predictive value of advanced PA metrics (e.g., quantile functional profiles), which capture the temporal complexity of PA time series and ultimately lead to more accurate survival predictions. Previous literature has indicated that PA is an important prognostic factor in ALS, with significant associations between PA and survival [14–16]. In general, ALS studies for endpoint development are often observational, and differences in design and patient populations make comparing predictive performance between studies challenging. Even in larger samples, the predictive capacity of such models tends to remain modest, and the concordance index (C-index) rarely exceeds 0.7.

The strengths of this study include the use of high-resolution PA data collected in free-living conditions over multiple days using the Beiwe smartphone application. This near-continuous data collection allows for a granular real-time examination of participant behavior with minimal participant burden. The statistical distributions introduced here offer a novel approach to analyzing continuous-time PA data in ALS research. They capture the substantial variability present both within and between days. Using quantile functions derived from the observed data to approximate daily PA behavior, the analysis does not require continuous step count monitoring 24 h a day.

Our study has several limitations, most notably the small sample size and the inclusion of relatively high-functioning ambulatory participants, which may limit generalizability to the broader population of ALS. We used a conservative inclusion criterion due to the large proportion of missing data in smartphone-collected physical activity measurements—an issue that can be magnified in neurodegenerative diseases that affect a person’s ability to use their phone. This conservative approach was adopted to increase the reliability of our exploratory findings. As a result, we analyzed only 37.6% of the total sample. Although this strategy helps ensure data quality, missing data remain a significant concern and may require novel statistical methods in future research. Additionally, among participants who died during follow-up, 90% died within 3 years; however, this is unlikely to compromise the observed statistical associations, as participants were followed for varying periods of time. The study may also be subject to selection bias because inclusion required participants to have at least ≥14 valid data days, which may have excluded individuals with more severe disease, lower adherence, or irregular device use. As a result, the findings may overrepresent more adherent or higher-functioning participants and may not be fully generalizable to the broader ALS population. To mimic a clinical trial screening period, we restricted our baseline analysis to ALSFRS-RSE measurements and the first 60 days of PA data; using additional data might improve survival predictions but may still be limited in ALS trials. We also focused solely on step counts, although other PA-related metrics may offer further insights. Finally, our models were limited to age, ALSFRS-RSE, and PA. We recognize the simplicity of these models, and future research could integrate PA data represented by quantile functional profiles into more robust predictive algorithms, such as those developed by Origent Data Sciences or ENCALS [17].

Recent findings indicate that gait alterations in ALS reflect both motor and cognitive dysfunction, which is very relevant to our results on smartphone-derived physical activity. Mild cognitive impairment has been associated with increased gait variability and the risk of falls [18], while the new classification of mild behavioral and neurocognitive impairment provides a framework for linking cognitive changes with the prognosis [19]. Our findings, showing that distributional representations of smartphone-based physical activity are associated with survival, complement this literature by suggesting that remote, quantitative mobility metrics may capture both motor and extra-motor aspects of the progression of ALS.

Future studies should include larger and more diverse cohorts of ALS and combine gait and physical activity measures with other smartphone-based digital biomarkers. In particular, voice analysis has shown sensitivity to bulbar dysfunction and overall disease severity [20, 21]. Integrating gait and physical activity measures with voice-based monitoring could enable a more comprehensive multimodal framework for remote assessment of ALS, improving both disease monitoring and risk stratification in future studies.

## Data Availability

The datasets generated and/or analyzed during the current study are available from the corresponding author on reasonable request. The datasets are not publicly available because they include sensitive information that may compromise participant privacy, and therefore are subject to ethical restrictions.
